# Radial neuropathy caused by intraneural leiomyoma: A case report

**DOI:** 10.1097/MD.0000000000020196

**Published:** 2020-05-29

**Authors:** Byung Chan Lee, Hyun Jin Kim, Yoon La Choi, Byung Joon Jeon, Duk Hyun Sung

**Affiliations:** a Department of Physical and Rehabilitation Medicine; b Department of Pathology and Translational genomics; c Department of Plastic Surgery, Sungkyunkwan University School of Medicine, Samsung Medical Center, Seoul, Republic of Korea.

**Keywords:** leiomyoma, magnetic resonance imaging, peripheral nervous system neoplasm, radial nerve

## Abstract

**Introduction:**

Leiomyoma of peripheral nerve is a rare condition characterized by neuropathy of affected nerve. We herein report a rare presentation of leiomyoma of radial nerve which presented with wrist drop.

**Patient concerns:**

A 37-year-old man visited our clinic with a history of sudden onset weakness of the wrist dorsiflexion/finger extension of the right side.

**Diagnosis:**

T2-weighted with fat saturation image of MRI demonstrated a well-defined, intra-neural, round mass of about 0.8 cm × 0.5 cm within the radial nerve. Excision of mass established the pathological diagnosis of intra-neural leiomyoma.

**Interventions:**

The patient underwent excision of mass and attached nerve tissue, followed his medial antebrachial nerve graft for repair of the defected radial nerve.

**Outcomes:**

As of the 1-year follow-up, no symptoms of recurrence have been observed. Also, the strength of wrist dorsiflexion improved to grade 4/5

**Conclusion:**

This rare case demonstrates the importance of MR imaging to differentiate intra-neural leiomyomas from other benign peripheral nerve sheath tumors. Surgical treatment plays an important role in the treatment of patient with intraneural leiomyoma with neurologic deficits.

## 1 Introduction

Tumors of the peripheral nerves are either neurogenic tumors or non-neurogenic tumors. Neurogenic tumors include the schwannoma, the neurofibroma, and the malignant peripheral nerve sheath tumor. Non-neurogenic tumors include lipoma, intraneural ganglions, lymphoma and intraneural epithelial or mesenchymal tumors.^[1]^ There have been a few reports of focal mono-neuropathies caused by peri- or intra-neural leiomyomas.^[2–6]^ We report a case of radial neuropathy caused by an intra-neural leiomyoma in a 37-year-old male who experienced sudden onset of weakness in his right upper extremity.

## 2 Case report

A 37-year-old man visited our clinic with a 6-month history of sudden onset weakness of the wrist dorsiflexion/finger extension of the right side. He reported that he experienced no pain at the time of the onset of weakness, but pain developed in the extensor side of the affected upper arm after 1 month. Under the Medical Research Council scale for grading muscle strength, volar-flexion of the right wrist joint was scored as 5/5, but strength of the right wrist dorsiflexion was decreased as 2/5, and radial deviation was observed at wrist dorsiflexion. He scored 5/5 on flexion, but 0/5 on extension of the right metacarpophalangeal joints. Strength of right forearm supination was decreased as 4/5. Tendon reflexes were normal at both biceps brachii, but were decreased at the right triceps brachii. Sensory examination revealed hypoesthesia at the dorsum of the radial side of the right hand.

**Figure 1:**
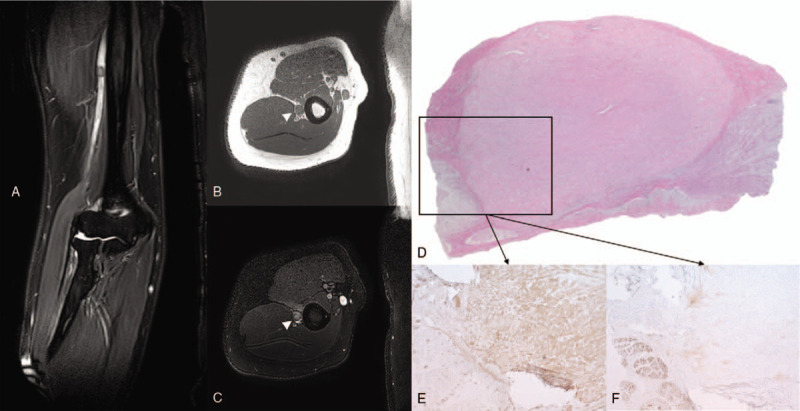
Right humerus MRI. T2-weighted with fat saturation image (A) showed a 0.8 cm × 0.5 cm size round mass within the radial nerve. Increased signal intensity and enlargement of the radial nerve below the radial groove of the right humerus was also noted. This mass exhibited an iso-intensity signal, and a well demarcated hypo-intense rim surrounded the mass in T1-weighted and T2-weighted images (B, C). Intraneural leiomyoma. Hematoxylin and eosin (H and E) stain (D) demonstrated fascicles of the radial nerve compressed at the periphery of the trunk by an intraneural tumor mass, and some axonal degeneration of the affected nerve fascicle was noted. Immunohistochemistry staining (E) showed diffuse smooth muscle actin (SMA) positivity in contrast to the S100 positive nerve fascicles shown in (F). MRI = magnetic resonance image.

Nerve conduction studies demonstrated that sensory nerve action potential and compound muscle action potential of the right radial nerve were not evoked. In the needle electromyography, spontaneous denervation potentials and neuropathic motor unit action potentials (MUAPs) on volition were observed in his right brachioradialis, extensor carpi radialis longus, and supinator. Denervation potentials were observed in his right extensor digitorum communis, extensor indicis proprius, and extensor carpi ulnaris, and no volitional MUAP was noted in these muscles. In the triceps, normal MUAPs were noted without denervation potentials. The physical examination and electro-diagnostic tests led to the suspicion that the radial nerve's pathologic lesion site resided at the right middle to distal humerus level. T2-weighted with fat saturation image of MRI demonstrated a well-defined, intra-neural, round mass of about 0.8 cm × 0.5 cm within the radial nerve. This mass exhibited an iso-intensity signal on the T1-weighted image and a slightly hyper-intense signal on the T2-weighted image as compared to adjacent muscles. Signal intensity was homogeneous in both T1- and T2-weighted images. On the enhanced T1-weighted image with fat saturation, the mass showed homogeneously mild enhancement. A well demarcated hypo-intense rim surrounding the mass was also noted in the T1-weighted and T2-weighted images (Fig. 1).

Surgical exploration revealed an approximately 0.8 cm × 0.5 cm-sized, firm, round mass that appeared to encircle the radial nerve below the radial groove of humerus. Palpation revealed that the radial nerve was indurated about 2 cm proximal and 4 cm distal to the mass. We attempted to completely separate the fascicles of the radial nerve from the mass, but it was not possible. Therefore, the tumor and attached radial nerve were excised together. Subsequently, the patient's medial antebrachial cutaneous nerve of the right arm was harvested and grafted for repair of the defected radial nerve. On the pathologic examination, the hematoxylin and eosin stain revealed fascicles of radial nerve compressed by an intra-neural tumor mass, and some axonal degeneration was noted. Bundles or fascicles of spindled cells with eosinophilic and possibly fibrillary cytoplasm were observed within the tumor mass. Immunohistochemistry staining was negative for S100 and desmin, but positive for smooth muscle actin, consistent with leiomyoma (Fig. 1).

After 3 months, the patient had improvement in the strength of wrist dorsiflexion to grade 4/5 after 12 months. Strength of extensor digitorum communis and extensor indicis proprius recovered to grade 3/5 and grade 1/5, respectively.

## 3 Discussion

A leiomyoma is a benign smooth muscle tumor that occurs in any organ wherever smooth muscle is present, and the most common forms occur in the internal organs, such as the uterus. An intra- or perineural leiomyoma causing focal neuropathy is extremely rare. There are few case reports detailing leiomyomas arising from the median nerve at the wrist level,^[2,4,7]^ from the median nerve at elbow level,^[5]^ and from the ulnar nerve at the level of epicondyle.^[3]^ A perineural leiomyoma causing tarsal tunnel syndrome has also been reported.^[6]^

MR imaging technique is important for differentiating intra or peri-neural tumors. Both neurofibromas and schwannomas, the most common peripheral nerve tumors, might exhibit high signal intensity in the periphery and low to intermediate signal intensity in the center on fluid-sensitive sequences with T2 contrast, the target sign. The target sign is caused by zonal architecture with more myxoid material peripherally and more fibrous tissue centrally.^[8]^ Intramuscular lesions may be surrounded by fat, a feature that can create the “split fat sign” on T1-weighted images. In contrast, a vascular leiomyoma within an extremity shows mixed areas of hyper- and iso-intensity to skeletal muscle on T2-weighted MR images, and these tumors also demonstrate a hypo-intense rim from the fibrous capsule surrounding the leiomyoma.^[9]^ In the present case, the intraneural leiomyoma showed homogeneous iso-intensity on the T1-weighted image and slight hyper-intensity on the T2-weighted image along with a well demarcated hypo-intense rim surrounding the mass in both the T1-weighted and T2-weighted images.

Additionally, the intraneural leiomyoma in the present case was encapsulated within the epineurium and adherent to the parent radial nerve so that complete dissection was difficult. We chose to sacrifice the parent nerve and harvest the patient's medial antebrachial cutaneous nerve for graft and repair of the defected radial nerve. No recurrence was noted at the 1-year follow-up visit, and the patient experienced some level of functional recovery.

Nerve repair techniques include autograft, allograft, synthetic conduit, and vein conduit. For small gaps up to 3 cm, the application of artificial conduits has the same success rate as nerve autograft repair, which results in recovery in up to 69% of cases. however, application of autologous nerve grafts is considered as more effective in nerve injury with a gap of more than 3 cm.^[10]^

Nerve autografts provide scaffold, including viable Schwann cells and neurotrophic factors, which play an important role in axon regeneration.^[11]^ Sural nerve, lateral antebrachial cutaneous nerve, and the anterior division of the medial antebrachial nerve were popular choices as a donor nerve.^[12]^

Secer and colleagues^[13]^ published a series of 2210 peripheral nerve injury and repairs in 2008. Their series included 255 radial nerves treated with autografting. M3 or better motor recovery was seen in 71.67% of radial nerve repairs. The mean time of follow up was 2.6 years.

Although intraneural leiomyomas are very rare, they can develop in peripheral nerves. Physicians should consider intraneural leiomyoma as a possible diagnosis in a case of a well-demarcated intraneural mass causing focal mono-neuropathy. Utilizing its characteristic signs on MR imaging (homogeneous internal signal intensity, absence of split fat sign or target sign, and presence of hypo-intensity rim around the mass in T1-weighted and T2-weighted images), physicians can differentiate intraneural leiomyomas from other benign PNSTs.

## Author contributions

**Conceptualization:** Yoon La Choi, Byung Joon Jeon, Duk Hyun Sung.

**Data curation:** Hyun Jin Kim, Yoon La Choi

**Methodology:** Byung Joon Jeon

**Supervision:** Duk Hyun Sung.

**Writing – original draft:** Byung Chan Lee

**Writing – review & editing:** Duk Hyun Sung
